# CO2 emissions and energy technologies in Western Europe

**DOI:** 10.1007/s13209-021-00234-8

**Published:** 2021-05-25

**Authors:** J. Barrera-Santana, Gustavo A. Marrero, Luis A. Puch, Antonia Díaz

**Affiliations:** 1 CEDESOG, Universidad de La Laguna, Santa Cruz de Tenerife, Spain; 2 Instituto Complutense de Análisis Económico, Universidad Complutense de Madrid, 28223 Madrid, Spain; 3 Universidad Carlos III de Madrid, Madrid, Spain

**Keywords:** CO2 emissions, Energy, Business cycles, Panel data, C23, Q43, Q5

## Abstract

In this paper, we investigate the path to the green transition in Europe. In so doing, we implement an empirical model of dynamic panel data on a sample of sixteen Western European countries over the period 1980 to 2019. The model is consistent with various features of neoclassical growth theory incorporating energy use. Our focus is on the short-run determinants of carbon emissions within that set of countries. We provide evidence that the relationship between economic activity and CO2 emissions is strong in economies where economic booms depend on energy-intensive sectors. Also, the mitigating role of renewable energy technologies is key when energy intensity rebounds. These circumstances may constitute a challenge for the climate transition goals targeted in the EU’s Recovery Plan, whose main objective at this very moment is to mitigate the economic and social impact of the coronavirus pandemic.

## Introduction

One of the more pressing issues in the international agenda is fighting global warming. Climate policy in the short-to-medium run requires a deep understanding of the connection between the emissions of greenhouse gases and economic activity. While energy intensity, i.e., energy consumed per unit of output, has been declining in most OECD economies in the last decades, it is apparent that there is substantial variation across countries [cf. Mulder and de Groot (2012) and Camarero et al. (2013)]. Also, the pattern of adoption of clean energy technologies among developed countries is very heterogeneous [cf. Apergis and Payne (2010) and Inglesi-Lotz (2016)]. Western European countries are not an exception to this general description. At the same time, this set of countries shares a lot in common among them in their production and energy technologies, particularly through trade. This circumstance makes useful to organize the relevant evidence on the path to the green transition in Europe, yet after various decades of climatic concern. We consider that such an assessment is key at this very moment when the ecological transition is at the center of the EU’s Recovery Plan to mitigate the economic and social impact of the coronavirus pandemic.

In this paper, we provide evidence on the complex interaction between CO2 emissions and aggregate economic activity. To do so, we estimate an empirical model of panel data on a sample of sixteen Western European (WE16) countries over the period 1980 to 2019, so just before the arrival of Covid-19. Our framework is one of the neoclassical growth theories, but our focus is on the economics of the business cycle fluctuations in this set of countries. The main hypothesis is that boom–bust dynamics might be a strong driver of CO2 emissions, and therefore, it might be important to incorporate cyclical considerations into climate policy. To identify the relevant comovements, we put together measures of GDP growth, the time variation in energy intensity, and the degree of advancement in the share of renewable energies. The ultimate goal is to provide an adequate measure of the income elasticity of CO2 emissions: the short-run within-country CO2–GDP elasticity. We aim at characterizing what is the role of the energy variables in the transmission of economic activity into climatic damage in the short-run, and on top of that, what is the role that renewable energies and energy efficiency might have been playing in that transmission channel in recent years.

Indeed, rapid improvement in the cost of renewable energies leads scholars to argue there is no dilemma between climate and the economy. However, the contribution to carbon concentrations in the atmosphere by some Western European countries continues to increase. Most countries exhibit reductions in the flow of per capita CO2 emissions between $-0.5$ and $-1.5$ per cent per annum, but the entire pool remains at a somewhat disappointing minus one per cent per year. At the same time, per capita GDP growth has been in most of the cases between 1.2 and 1.8 per cent per year over the period, reaching a 1.6 per cent growth in the entire pool. As we illustrate, and this is the key finding, the heterogeneity within this group of rich countries in its CO2-income path mostly comes from differences in energy intensity, and not that much because of variation in the potential mitigating role of renewables.

In this respect, there is very relevant information in the evolution of the cross section data across countries, as well as in the time series country by country. First, we provide a thorough description of the available data on carbon emissions, energy use, and the economic activity, both in the pool of countries and in the time series. We observe that the small long-run CO2–GDP correlation in the data can be partly explained by a high heterogeneity in the short-run within-country correlation between CO2 and GDP growth. Two key variables seem to account for such a heterogeneity: first, the CO2 emissions inertia, that is, the fact that countries starting with higher levels of emissions may reduce their emissions more (or not) and second, the role of energy variables, both the differences in energy intensity and in the share of renewable energies in the primary energy mix.

We use a theoretical framework that helps us to specify an empirical model of the panel data in order to extract the relevant within-country evidence. We build upon a strand in the literature of macroeconomic models of energy use. In those models, energy is an essential input that combines with physical capital into a putty-clay technology as in Atkeson and Kehoe (1999) and Díaz and Puch (2004). The key issue is the energy requirement which is fixed and cannot be changed once capital is installed, but at the same time, there are various mechanisms to substitute energy with more energy-efficient capital in the medium-to-long run. Thus, in the short-run there is a close to zero elasticity between the capital–labor composite and energy, while most of the potential for adjustment in the energy aggregates takes time-to-be-built. We think this is a focal point over which climate policy has to step in, and several attempts have been made to incorporate a green transition of this form in such a macroeconomic framework for policy purposes, as in Hassler et al. (2019) or Díaz and Puch (2013, 2019). Here, we are closer to the reduced-form approach in Marrero (2010), Díaz et al. (2019) and Díaz et al. (2020) that builds in this tradition of macroeconomic models, and augmented to incorporate the dynamics of CO2 emissions as in Stokey (1998) or Golosov et al. (2014).

With this theoretical background, we propose an empirical model for energy, CO2 emissions, and the macroeconomy which we consider adequate to be implemented and estimated for our sample of developed countries. The goal is to characterize the existing heterogeneity of the short-run within-country CO2–GDP elasticity. A proper understanding of this elasticity is needed to assess the interaction between CO2 emissions and economic activity for policy purposes in Western Europe.

Climate policy has often a focus on extreme warming scenarios and aggressive action (see Weitzman 2009, among others). Rather, our view here stresses potential intermediate stages for corrections. There is the important issue of energy price and technical change uncertainties, which can be seen as barriers to the adoption of energy-efficient technologies. These operate in part at business cycle frequencies. Thus, there might be an important role for tax-based (and subsidies) stabilization (as a complement to cap and trade), not sufficiently studied in the environmental policy literature. We illustrate that cyclical adjustment mechanisms could be used to smooth the costs of climate policy over the business cycle to given emissions targets, in line with Metcalf (2020). Finally, our approach highlights a bottom-up design according to which regional progresses in regulations expand and integrate to other countries (see Battaglini and Harstad 2016). We believe that the evidence in this paper can contribute to climate policy programs in Western Europe, and particularly, at this very moment, in connection with the EU’s recovery plan post-Covid-19.

We find that an important part of the within-country correlation between CO2 and GDP growth is driven by factors that are common to all countries but time variant. We interpret this evidence as one of the carbon emissions being sensitive to the common Western European business cycle. At the same time, changes in energy intensity over the panel ($\Delta EI$) turn out to be the key variable to account for CO2 emissions growth, once we control for the common business cycle and other factors. Precisely, an improvement in one standard deviation of $\Delta EI$ is associated with a reduction in nearly two-thirds of a standard deviation in CO2 annual growth. Notwithstanding, as indicated above, a question we address is whether the path of CO2 emissions for countries at different positions of their energy technologies is more or less responsive to business cycle fluctuations. We find evidence that it is not GDP growth per se which brings about additional CO2 emissions. Rather, it is GDP growth whenever energy intensity is high that triggers the alarms. We take this interaction as a proxy for the green (or not) transition dynamics. A transition for which the role of changes in renewable energies uses has a direct and highly significant effect on the reduction of CO2 emissions, but it has a very moderate effect on the short-run within-country CO2–GDP elasticities. The main implication of all these findings is the absolute priority for policies that contribute to reaching conditional convergence in energy intensity standards across Western European countries.

Finally, we present four alternative specifications for the within-country, short-run estimates we compute. These are intended to address (i) the particular role of different countries, (ii) the precise role of boom–bust dynamics, (iii) the precise role of different renewable energies, and (iv) some account for the role of imports. We examine these alternative specifications, and we show that in all of the cases, our benchmark regression coefficients remain plausible. Notice that boom–bust dynamics are often associated with expectation-driven cycles in credit or housing markets and might go beyond benchmark economic fluctuations for some particular countries.^1^ As a consequence, boom–bust cycles can have important asymmetric effects between economic shocks, energy intensity, and carbon emissions that we also investigate in this paper.

The paper is organized as follows. Section 2 presents some preliminary evidence on the linkages between economic growth and CO2 emissions, and how those linkages operate through the energy technologies. Section 3 proposes a theoretical framework to relate CO2 emissions with the production and the energy technology that serves as a building block for the empirical model. Then, we discuss the empirical implementation of the model in Sect. 3.2, and Sect. 4 reports the main estimation results. Section 5 discusses various robustness checks, and the last section concludes.

## The interaction between CO2 emissions and economic activity in Western Europe

We start with descriptive evidence on the evolution of per capita CO2 emissions and per capita GDP in our set of 16 Western European countries between 1980 and 2019. We compute the correlations between the growth rates of these variables as a proxy of the short-run CO2–GDP elasticity. Then, we introduce a dynamic aspect with respect to the generation of CO2 emissions, showing that the emissions intensity is persistent. We further illustrate that the assumption of common elasticities between countries makes no sense in our sample. This motivates incorporating the role of the energy technologies to understand the heterogeneity in the CO2–GDP elasticity across these countries.

In “Appendix A,” we document the data series we use (Table 9). Polluting emissions are measured in thousands of tonnes of CO2, whereas GDP is purchasing power parity (PPP) adjusted to 2015 thousands of USD. Population is measured in millions. All these data are taken from the most recent releases of the IEA (2020). Thus, our results provide up-to-date insights on the interactions between CO2, GDP, and the energy variables, in this set of Western European countries that share so much of their production and energy technologies.

### Preliminary evidence

Table 10 in Appendix reports the main descriptive statistics for per capita CO2 emissions and GDP. We can summarize this preliminary evidence as follows. First, most countries exhibit reductions in per capita CO2 emissions, which go from $-0.5\%$ (Italy, the Netherlands, Norway, or Switzerland are in this side) to $-1.5\%$ per annum. (Belgium, Finland, or Germany are more in this other side.) The virtuous cases are Denmark and Sweden, which have been doing slightly better than the UK or France in the emissions dimension. These four countries started with big levels of per capita CO2 emissions in 1980. Portugal and Greece did not do well, and both countries increased their CO2 emissions over the period. Austria, Ireland, and Spain appear to be below the standards as well, with emissions levels very similar to those in 1980. All in all, the annual CO2 emissions reduction for the 16 countries falls short of one per cent per year. At the same time, per capita GDP growth has been for most of the countries between 1.2 and 1.8%, with Portugal and the UK in the more favorable side and France in the other side of this range. Ireland is a top outlier in this metric, showing an annual growth of about 3.9% over the period, whereas Greece, Italy, and Switzerland have experienced annual growth rates below 1%. In the pool, average GDP growth per annum is close to 1.6%.

A simple inspection of the data suggests that the long-run variation in per capita CO2 emissions is negatively correlated with their initial levels (Fig. 3a). However, that correlation is practically non-existent with the average per capita GDP growth (Fig. 3b). Thus, in a long-run cross-country comparison, the evidence of convergence in CO2 emissions is clear: Countries with higher (lower) levels of emissions in 1980 have reduced emissions more (less) between 1980 and 2019. Notwithstanding, we need a closer revision of the data to understand the absence of long-run correlation between per capita CO2 and GDP in our sample. We will return to the notion of convergence later, but we focus next on this second element.

**Fig. 1:**
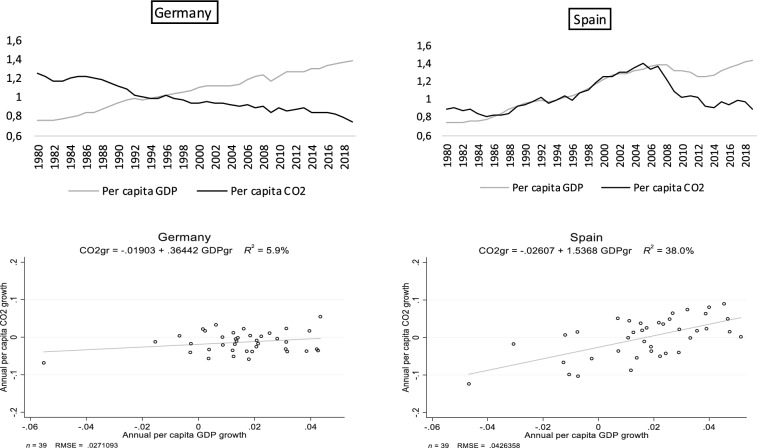
Spain versus Germany. **a**, **b** Per capita GDP and per capita CO2 indexes between 1980 and 2019 (index $1994=1$). **c**, **d** Heterogeneity in CO2–GDP

### The CO2 emissions–GDP relationship

The small long-run CO2–GDP correlation observed above can be partly explained by a high heterogeneity in the short-run within-country correlation between CO2 and GDP growth. Figure 1 depicts the annual evolution of per capita CO2 and per capita GDP (top panel of the graph), as well as the scatter plots of their annual growth rates (bottom panel), for two selected countries in our sample: Germany and Spain.

The plots in the top panel suggest that these countries have experienced very different growth stories over the sample. In Germany, per capita GDP shows a relatively stable growth path (with around 1.5% growth per year). A similar stability occurs for its per capita CO2 emissions, albeit this series decreases smoothly at a rate of 1.3% per year. The situation is very different for Spain, with per capita GDP growth associated with a boom and bust pattern, and CO2 emissions evolving pairwise until the 2008 crisis, when, by now, a transitory decoupling is observed in the comovement between these two variables.

With respect to the scatter plots, the case of Germany is one that exhibits very low CO2 emissions–GDP elasticity, about 0.364 in our sample, whereas Spain displays the less favorable response, well above one, and up to 1.54. Clearly, in Spain, over the last nearly 40 years, it has been difficult to observe significant GDP growth without emissions growth. The opposite is observed in the case of Germany, where few observations display significant per capita CO2 emissions growth. This well-known fact is quite disappointing for Spain as a start, and very much related to the construction boom of the late 90s until the Great Recession [see, for instance, Gutiérrez et al. (2011)].^2^

Indeed, we examine this important evidence for the fourteen other countries in our database, which are reported in “Appendix B.” Beyond the illustration above for Germany vs Spain, the first observation is the important heterogeneity in trends and CO2–GDP slopes, the latter ranging from 0.17 in Switzerland to, as already mentioned, 1.54 in Spain. The more pessimistic observation is that slopes bigger than one occur not in a few cases (see Fig. 5 in “Appendix”). These are: (Belgium, see below), Denmark, Italy, Norway, Portugal, and Spain. A number of other countries are not far from that observation either, with elasticities above 0.70, as it is the case for Austria, France, or the UK. Therefore, nine out of sixteen closely related developed countries, all of them sharing quite a lot of their production and energy technologies, exhibit a very high income elasticity of emissions according to this very simple measure. This is not good news. Finally, going back to GDP and CO2 trends (Fig. 4 in “Appendix”), we observe that some countries behave like Germany (as France, the Netherlands, Sweden, Switzerland, or the UK), others more like Spain (as Austria, Greece, Italy, or Portugal), while another group stays somewhere in the middle group (as Belgium, Denmark, Finland, Ireland, or Norway).^3^

### CO2 emissions inertia

One important issue in our descriptive approach at this point is whether or not the income elasticity of emissions varies when controlling for the CO2 emissions inertia. Notice that according to this alternative, we move from the linear regression:

2.1 $$\begin{aligned} \Delta \ln (CO2/POP)_t = \beta _0 + \delta _0\,\Delta \ln (GDP/POP)_t + {\widetilde{\varepsilon }}_t, \end{aligned}$$

corresponding to the representation in Fig. 1, toward:

2.2 $$\begin{aligned} \Delta \ln (CO2/POP)_t= & {} \beta _0 + \delta _1\,\Delta \ln (GDP/POP)_t\nonumber \\&+ \beta _1 \ln (CO2/POP)_{t-1} + \varepsilon _t, \end{aligned}$$

for each and every country in the sample. Equation (2.2) expresses $\ln (CO2/POP)_t$ as a function of $(1+\beta _1)\,\ln (CO2/POP)_{t-1},$ and therefore, a positive $\beta _1$ implies a strong inertia in the CO2 emissions path (that can be found outside a convergence growth trajectory). On the contrary, a negative $\beta _1$ implies that the effect of a shock on emissions disappears as time goes by, and consequently, we have some form of unconditional convergence in CO2 emissions in the sample.

Table 1 compares the estimated $\delta _{0}$ with $\delta _{1}$ and shows also the estimated dynamic term $\beta _{1}$ for each country. The results suggest that such a convergence is the general pattern except maybe for Italy (${\widehat{\beta }}_1 = 0.051$) and the UK (${\widehat{\beta }}_1 =0.048$). Remember that Italy is one of the paradigmatic low growth cases (included in the emissions group of Spain, say), whereas the UK showed up as one of the highest growth examples (included in the emissions group of Germany). With respect to emission-GDP elasticities, there are some adjustments, but most of them are not statistically significant. Only for Norway, Portugal, or the UK, the elasticity (comparing ${\widehat{\delta }}_0$ to ${\widehat{\delta }}_1$) exhibits a decline beyond $-10\%$. But, in general, most of the countries exhibit small changes in the CO2–GDP elasticity once we control for the inertia in CO2 emissions. For instance, the nine countries indicated above as showing a CO2–GDP elasticity greater than 0.7 are now the same, regardless we include or not the inertia term. This comparison may suggest that the inertia of CO2 emissions is not key in explaining the observed heterogeneity in the CO2–GDP elasticity in our sample. However, we illustrate below that is quite imprecise to look to the income elasticity of emissions without looking to the evolution of the energy technology over the sample.

**Table 1:** Income elasticity of per capita CO2 emissions growth and CO2 inertia in WE16 countries

CO2pc growth							
	(1)		(2)				
	GDPpc Growth		GDPpc Growth		L1.CO2pc		
	Elasticity	Std. Error	Elasticity	Std. Error	Elasticity	Std. Error	$R^2$
Austria	0.8508	0.4836	0.7939	0.4297	$-$ 0.1087	0.0662	0.1264
Belgium	1.0318	0.3729	1.1690	0.3607	$-$ 0.0848	0.0484	0.1773
Denmark	1.2448	0.6609	1.2581	0.6725	$-$ 0.0089	0.0538	0.0658
Finland	0.4663	0.2710	0.5619	0.2966	$-$ 0.1531	0.0919	0.0869
France	0.7220	0.3381	0.8403	0.3239	$-$ 0.0988	0.0471	0.1943
Germany	0.3644	0.2721	0.3901	0.2782	$-$ 0.0109	0.0333	0.0671
Greece	0.5192	0.1951	0.5680	0.1903	$-$ 0.0677	0.0306	0.2460
Ireland	0.3906	0.1498	0.3907	0.1506	0.0003	0.0416	0.1876
Italy	1.3498	0.1919	1.3895	0.2004	0.0510	0.0360	0.5990
Netherlands	0.3308	0.2345	0.3579	0.2189	$-$ 0.0630	0.1222	0.0419
Norway	1.0232	0.5238	0.9003	0.6347	$-$ 0.0827	0.1446	0.0706
Portugal	1.2544	0.4420	1.1054	0.4127	$-$ 0.0531	0.0347	0.2500
Spain	1.5368	0.3182	1.5341	0.3311	$-$ 0.0014	0.0430	0.3802
Sweden	0.3896	0.3703	0.3950	0.4049	$-$ 0.0030	0.0380	0.0268
Switzerland	0.1697	0.4068	0.1749	0.4178	$-$ 0.0091	0.0485	0.0044
UK	0.8174	0.2791	0.6997	0.3084	0.0479	0.0259	0.2347

In model (1), we regress growth in per capita CO2 emissions over per capita GDP growth. Model (2) extends model (1) by including the 1-year lagged effect of per capita CO2 emissions (Lag-CO2pc). Note that some elasticities may be affected for the year 2009 outlier, as indicated in the main text

### CO2 emissions and the energy variables.

The question is now whether the evolution of energy use over time and the role of renewable energies can contribute to account for the observed heterogeneity in the data. We focus on the role of the changes in energy intensity (EI) and in the share of renewables. EI is measured in kToe/BillionUSD, and the share of renewables is between zero and one again, the data from the IEA (2020). The changes in these energy variables are measured in differences of kToe/BillionUSD, and in percentage points (p.p.), respectively.

**Fig. 2:**
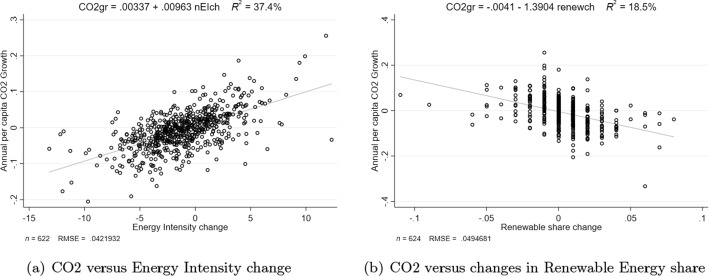
**a** CO2 versus energy intensity change. **b** CO2 versus changes in renewable energy share. Both figures are based on pooled WE16 countries between 1980 and 2019. Note two outliers have been dropped: IRL 2015 and NOR 2019

**Fig. 3:**
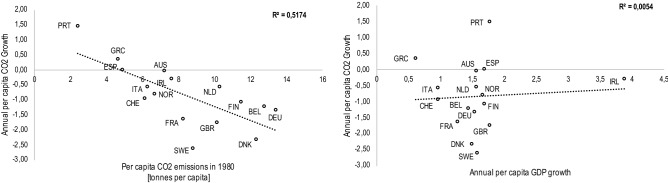
Long-run variation in per capita CO2 growth...

Figure 2 shows for the entire pool of data, on the one hand, the joint evolution of CO2 emissions growth and energy intensity (EI) in the sample (left panel) and, on the other hand, the potential role of changes in the share of renewable energies over time (right panel). Both correlations display the expected signs. First, there is a positive and significant correlation between increases in energy intensity and CO2 emissions growth in the pool of 16 countries. The slope in the scatter plot is 0.01. Most of our country-years observations show values that range from $-10$ to +10 kToe/BillionUSD as absolute changes of EI. These EI changes are associated with average per capita CO2 emissions annual growth rates between $-0.2\%$ and 0.2%, respectively. Correspondingly, the correlation between CO2 emissions growth and the changes in the share of renewable energies in the primary energy mix is negative, with an estimated slope of $-1.39$. In this case, the most frequent changes of the share of renewables range from $-5$ p.p. to +5 p.p. Notice that the measure of the changes from one period to the other in the renewable energies’ share is discrete, as these are the data reported by the IEA.

Finally, the illustration in the pool (rather than country by country) can be further structured when we distinguish between either high and low energy intensity observations, or high and low renewable energy share observations. Precisely, Fig. 6 in “Appendix C” splits the sample into country-years observations of energy intensity above and below the median for the entire pool, and this cutoff is 94 kToe/BillionUSD. In the high energy intensity case, the estimated slope of the CO2–GDP correlation is 0.815, whereas for the low energy intensity observations the slope is 0.524. Correspondingly, Fig. 7 in “Appendix C” splits the sample into country-year observations of renewable shares above and below the median. (For the entire pool, this cutoff is 8%.) The finding is that the CO2–GDP slope goes from 0.735 for the high renewable share observations to 0.605 for the low renewable share observations, a somewhat narrower difference than for the EI case.

Now, we are in a better position to characterize the combined information that the cross section and the time series provide for this group of seemingly much related countries. We will pursue this analysis through a panel data approach. Prior to obtain some panel data estimates, we present next a theoretical background that aims to bring some discipline to the empirical model.

## Theoretical background and the empirical model

### A simple theory of the link energy, emissions, and economic activity

In light of the preliminary evidence above, our goal is to establish an empirical relationship between CO2 emissions and the joint evolution of economic activity and energy use. We build upon the tradition of DICE (*Dynamic Integrated Climate-Economy*) models, and more precisely on the technological assumptions in Brock and Taylor (2005, 2010) and our previous work in Alvarez et al. (2005), and recently Díaz et al. (2019) or Díaz et al. (2020), to establish that relationship. The starting point is to consider a neoclassical production function augmented with an aggregate of energy use, $E_t$. We assume that production (per unit of labor, $L_t$), requires capital and energy (whatever the source) in the following way:

$$\begin{aligned} y_{t} = \left\{ \begin{array}{ll} {\widetilde{A}}_t\,k_{t}^{\alpha }\,e_{t}^{\theta }, &{} \hbox {if }e_{t}= v_{t}\,k_t; \\ 0, &{} \hbox {otherwise,} \end{array} \right. \end{aligned}$$

where $v_t$ is a technological (energy saving) index of the unit of capital [cf. Díaz and Puch (2019) for such an environment at the plant level], and ${\widetilde{A}}_t$ is an unadjusted measure of total factor productivity. Notice that we can write the production function (per worker) as:

3.1 $$\begin{aligned} y_t = A_t \left( \frac{e_t}{y_t}\right) ^{\frac{\alpha +\theta }{1-\alpha -\theta }}, \quad \text{ where } A_t = \left( {\widetilde{A}}_t\,v_t^{-\alpha } \right) ^{\frac{1}{1-\alpha -\theta }}. \end{aligned}$$

To make explicit the different sources of energy and, therefore, the energy mix, we specify carbon emissions in line with Stokey (1998).^4^ We assume that we can express the flow of CO2 emissions:

$$\begin{aligned} P_t = {\widetilde{E}}_t^{\phi }\,Y_t^{\varphi }, \end{aligned}$$

where now, ${\widetilde{E}}_t$ is counting energy in units of CO2 emissions, whereas $E_t$ in the production technology is expressed in units of energy. We do not need to be explicit on how the different energy technologies enter in the energy aggregate, $E_t,$ or the emissions’ generating process, ${\widetilde{E}}_t$ [see, for instance, Díaz et al. (2019), based on a preliminary version of Hassler et al. (2020)]. We do not need either to specify how the climatic damage is built from the flow of CO2 emissions, $P_t,$ in every period *t*,  [see, for instance, Golosov et al. (2014)]. We adopt the simplifying assumptions that there is some form of imperfect substitution between the different energy technologies both in production and in carbon emissions, on the one hand, and that the feedback from climate damage to the economy operates, on the other hand, by diminishing total factor productivity in the long run. This later assumption implies the feedback occurs well beyond the short-run scope of our empirical implementation.

Using (3.1), we can rewrite the flow of CO2 emissions as:

$$\begin{aligned} P_t = \frac{{\widetilde{E}}_t}{E_t}^{\phi }\,{E_t}^{\phi }\, \left[ L_t\,A_t\,\left( \frac{{E}_t}{Y_t} \right) ^{\frac{\alpha +\theta }{1-\alpha -\theta }} \right] ^{\varphi }. \end{aligned}$$

Finally, taking into account the energy requirement in the production technology, $E_t = v_t\,K_t,$ we can fully recover a parameterized version of this specification in the form:

3.2 $$\begin{aligned} P_t = \left( \frac{{\widetilde{E}}_t}{E_t}\right) ^{\phi }\, {\widetilde{A}}_t^{\gamma _1}\,v_t^{\gamma _2}\,Y_t^{\gamma _3} \,L_t{\gamma _4}\,\left( \frac{{E}_t}{Y_t} \right) ^{\gamma _5}. \end{aligned}$$

In this expression, the energy mix, ${\widetilde{E}}_t/E_t$, the energy intensity, $E_t/Y_t,$ and the aggregate economic activity, $Y_t,$ are made explicit, whereas the inertia of the model is embedded in both forms of technical progress we consider, that is, neutral technical progress, ${\widetilde{A}}_t,$ and the energy saving technical change index, $v_t.$ We assume, therefore, that technical change in the state of the energy technology in the short run can be summarized in part into $P_{t-1}$ through the process of carbon dynamics. Moreover, the reduced form specification of the state of the aggregate technology above can be made consistent with crossed effects of economic activity with energy intensity and the energy mix.

The variables selected with this theoretical background are based on well-established models in existing literature, following Brock and Taylor (2010) or Marrero (2010), and up to Díaz et al. (2020) as indicated above. It could be argued, though, that there are omitted variables. However, it is important to notice that the cross-sectional dimension we are considering is short, and that the selected set of countries share in common a lot of the institutional and regulatory framework. Therefore, we believe that the dynamic panel data framework with fixed effects we propose next, based on the production and emissions technologies we have specified in this section, is adequate to provide measurement of the short-run within-country effects we are looking for.

### The empirical model

We use annual data, and we consider either growth rates or annual changes of the relevant variables. Thus, our approach is more business cycle oriented than long-run growth based. This is motivated because we want to characterize the existing heterogeneity of the short-run within-country CO2–GDP elasticity. An adequate understanding of this elasticity is needed to assess the interaction between CO2 emissions and economic activity for policy purposes in Western Europe.

From the previous assumptions and with some further parameterization suited for these data [cf. Marrero (2010), Díaz et al. (2019, 2020)], we specify a version of Eq. (3.2) linearized:

3.3 $$\begin{aligned} \Delta \ln P_{i,t}= & {} \beta _0 + C_i + T_t + \beta _1 \, \ln P_{i,t-1} + \beta _2 \, \Delta \ln Y_{i,t} + \beta _3 \, \Delta EI_{i,t} \nonumber \\&+ \beta _4 \, \Delta R_{i,t} + \varepsilon _{i,t}, \end{aligned}$$

where $\Delta \ln P_{i,t}$ denotes per capita CO2 emissions annual growth; $C_i$ is a country-fixed effect that captures the long-run (unobservable) differences across countries; $T_t$ represents a time-fixed effect that captures the global business cycle effects and other global shocks that may be jointly driving emissions and economic activity in our sample; $P_{i,t-1}$ accounts for a one-period lag in per capita CO2 emissions (inertia or convergence term); $\Delta \ln Y_{i,t}$ is per capita GDP annual growth; $\Delta EI_{i,t}$ denotes the annual change in energy intensity; and $\Delta R_{i,t}$ represents the change in the share of renewables, which captures in a very parsimonious way the main source of variation in carbon intensity of energy use. Finally, $\varepsilon _{i,t}$ is a mean zero and constant variance $\sigma ^2$ innovation to this data generation process.

Notice that reverse causality (that is, whether $\Delta \ln P_{i,t}$ causes $\Delta \ln Y_{i,t}$) is not relevant in our application as it associates with a long-run feature of the data that goes from climatic damage to neutral progress as in Golosov et al. (2014), and the cross-sectional dimension we consider is short. In any case, we explicitly explore this issue below. Also, institutional and regulatory variables exhibit limited time variability in our sample for the set of countries we consider, and therefore, those potential treatment variables should be captured within the country-specific fixed effect. Moreover, we found that adding those variables brings loss of efficiency in the estimator due to potential correlation with the fixed effect.^5^ Finally, global elements of technical change, which are expected to be common across the set of countries in our sample, are expected to be captured by the time-specific fixed effect.

Under these circumstances, the key parameter is the elasticity $\beta _2$, which should be interpreted as an average within-country CO2–GDP annual elasticity. Thus, it can be compared with parameters $\delta _{0}$ and $\delta _{1}$ in Eqs. (2.1) and (2.2), and thus, the estimates in Table 1. The parameter $\beta _{1}$, which is expected to be negative, is associated with the conditional convergence speed of CO2 emissions in our sample.^6^ As we show next, the negative sign of this elasticity is confirmed in the panel regressions, whereas it was not always present in the country by country regressions. The estimated $\beta _3$ and $\beta _4$ denote the direct impact of the energy elements on the CO2 emissions. Since the shares of renewables and non-renewables add up to one, the $\beta _4$ coefficient measures the effect of a change in the renewables share with respect to the change in fossil fuels. For a better quantitative assessment of these relationships, the variables are scaled in such a way the estimated $\beta _{3}$ and $\beta _{4}$ represent the effect of a one standard deviation change over the annual emissions growth rate.

In the baseline specification, we assume $\beta _{2}$ to be constant across countries. However, as discussed above, this is an unrealistic assumption. We extend equation (3.3) including two interaction terms between $\Delta \ln Y_{i,t}$ and the lagged levels of the energy variables, say: $\beta _{21}\,EI_{i,t-1}\,\Delta \ln Y_{i,t}$, and $\beta _{22}\,R_{i,t-1}\,\Delta \ln Y_{i,t}$. Thus, we are allowing the short-run within-country elasticity between per capita CO2 emissions and GDP to be country-and-yearly-specific, as it depends on the lagged levels of energy intensity and the lagged share of renewables, that is: $\beta _{2}+\beta _{21}\,EI_{i,t-1}+\beta _{22}\,R_{i,t-1}$. As we will show below, in all the specifications considered $\beta _{22}$ is not statistically different from zero, and therefore, we will focus on the CO2–GDP elasticity as a function of the position in energy intensity: $\beta _{2}+\beta _{21}EI_{i,t-1}$.

### Econometric issues

In addition to exploiting the entire panel information of these data, our specification is convenient for at least two reasons. First, because it controls for time-varying and cross-country fixed and unobserved heterogeneity. Not considering these sources of heterogeneity may result in seriously biased estimates when those sources of heterogeneity exist (Hsiao (1986)). This feature of the data will be illustrated in Table 2 when comparing regressions (1), (2), and (3), showing that the estimated coefficients of $\beta _{1}$ and $\beta _{2}$ change with the inclusion of the fixed effects $C_{i}$ and $T_{t}$ in the model. Second, the estimated parameters represent what we actually want to measure: the (average) within-country and short-run partial correlations.

This specification does not guarantee unbiased estimations of $\beta _{1},$ though. Actually, ${\widehat{\beta }} _{1}$ is expected to be downward biased, as far as the estimate from the pool-OLS would be upward bias [again, Hsiao (1986)]. As we will further discuss in the next section (again, Table 2), the inclusion of energy variables in the model seems to reduce this potential bias. However, estimated results of $\beta _{2}$ could still be valid if $E[\Delta \ln Y_{i,t}\varepsilon _{i,t}]=0$ in equation (3.3), and in its extended version including crossed terms. Roughly speaking, this condition is likely to be satisfied if, first, $\Delta \ln Y_{i,t}$ is weakly correlated with $\ln P_{i,t-1}$, and second, there is no reverse causality in our sample (i.e., $\Delta \ln P_{i,t}$ does not cause $\Delta \ln Y_{i,t}$). With respect to the first condition, we already obtained the evidence of a moderate explanatory power of lagged emissions in Table 1, as discussed above. Moreover, the linear correlation coefficient between $\Delta \ln Y_{i,t}$ and $\ln P_{i,t-1}$ is just 0.050 (non-significant) for the entire pool, and nearly the same, 0.048 (non-significant), for those variables controlled by the fixed effects (i.e., the within-country and within-year correlation). Regarding the second aspect, it has been argued in Sect. 3.1 that the effect on GDP from climate damage through productivity as in Golosov et al. (2014) is fundamentally forward looking. The environmental damage would end up affecting total factor productivity through its effect on, for instance, health and then on human capital, but this mechanism will not operate in the short run.

Nevertheless, we take the endogeneity concern more seriously and perform endogeneity tests to every right-hand side variable included in equation (3.3), and when extended with the crossed terms. We follow the three-step procedure proposed by Wooldridge (2002). First, an OLS regression is estimated drawing on the lagged levels of the dependent variable (i.e., per capita CO2 emissions), controlling by country- and time-fixed effects. Second, the residuals of this regression are included in our main models as an exogenous variable. Finally, we conduct a post-estimation Wald test on the estimates corresponding to the residual term under the null hypothesis that such parameter is equal to zero. Rejecting the null hypothesis should raise concerns about endogeneity in the models. In our case, the test fails to reject the null hypothesis (p value $= 0.30$) which is an undoubtable symptom that endogeneity is not an important issue in the sample. The homogeneity of our data (specifically, a strongly balanced panel of Western European countries, starting from 1980) is clearly helping to reduce endogeneity problems.

The usual, mechanical, way to proceed when estimating the dynamic panel model is to use an instrumental variable approach. In the absence of external instruments, the alternative is to use internal instruments (i.e., lagged value of the endogenous variable and of the regressors).^7^ We have used one or two lagged levels of the variables as instruments, and we have obtained similar estimation results, but with the inconvenient that the Hansen test of overidentifying restriction fails in several specifications. The common alternative of the system-GMM (Arellano and Bover 1995), which uses a larger set of instruments, is specially designed for a large cross section in comparison with the time dimension, which is the opposite to our sample. In our case, we always have overfitting problems, even when using any method to reduce the number of instruments (Roodman 2009), hence system-GMM estimations are strongly inefficient in our case.^8^ For all that, an instrumental variable approach, in the absence of a good exogenous instrument (the most common situation in these macroeconomic models) and in the presence of exogenous (statistically speaking) regressors, would generate estimation problems, and using a pool-OLS with fixed effects (country and year) would be a more convenient and conservative strategy (see Bun and Sarafidis 2015). Our estimation results in the following section are based on this latter approach.

Notwithstanding, there is the important issue of time-variant unobserved heterogeneity that cannot be addressed with fixed effects. For instance, one may think of differences in regulation between the north and the south along the sample. We believe that the structural part of those differences must be channeled through the energy technologies, precisely, in the form of differences in energy intensity and in the share of renewables in the primary energy supply. On top of that, those differences (in regulation) related to the energy dimension are hard to observe at the panel frequency. Consequently, as we do not include them into the model, we assume that they are, if temporary and therefore unchanneled through the energy variables, incorporated to the residuals. Whenever this part incorporated in the residuals is small (notice the high $R^2$ of the regressions in the following section), and it is uncorrelated with energy aspects, its omission should not be affecting the estimation of our key coefficients.

## Estimation results

For the whole sample, we analyze first the within-country short-run relationship between CO2 emissions and the changes in economic activity levels. Then, we move toward the energy variables and analyze the potential for the changes in energy intensity and in the share of renewable energy to modify the patterns of CO2 emissions growth in the panel data. Finally, we examine the consequences of various interactions between GDP growth and lagged energy variables (in levels) that we take as a proxy for the green (or not) transition dynamics.

### Emissions, energy, and the business cycle

As indicated above, our specification involves emissions growth and the one-period lagged level of emissions. Table 2 reports the estimates of pool-OLS with country and temporal fixed effects. For illustrative purposes, we present the results in the following sequence. First, we estimate equation (3.3), but not including the interaction terms and, for comparative purposes with results in Table 1, at first we consider alternative assumptions related to the inclusion of country and temporal fixed effects and energy variables.

Column (1) in Table 2 shows the results for the entire pool of data, excluding fixed effects or the role of energy variables. Thus, the estimated results are directly comparable with the estimations from Eq. (2.2) on the preliminary evidence. Columns (2) and (3) add country- and year-fixed effects sequentially. It is worth mentioning the important changes in the estimations of $\beta _{1}$ and $\beta _{2}$ when including those fixed effects. Notice that constant can be dropped for country-fixed effect. As discussed above, not including both the country- and the year-fixed effects would bias the estimated parameters. Also, as we expected, in all of the cases we show a negative correlation between lagged CO2 and CO2 emissions growth (i.e., the convergence property), but the size of $\beta _{1}$ increases (in absolute value) when including the country- and the temporal-fixed effects. Therefore, this coefficient reflects now conditional convergence instead of absolute convergence.

**Table 2:** Panel data estimates: CO2 emissions, growth, and energy

	CO2pc growth					
	(1)	(2)	(3)	(4)	(5)	(6)
Lag-CO2pc emissions	$-$ 0.0227***	$-$ 0.0367***	$-$ 0.0888***	$-$ 0.0422***	$-$ 0.0845***	$-$ 0.0449***
	(0.00772)	(0.0140)	(0.0169)	(0.0127)	(0.0148)	(0.0123)
GDPpc growth	0.652***	0.683***	0.434***	0.813***	0.392***	0.754***
	(0.0937)	(0.0956)	(0.121)	(0.109)	(0.109)	(0.103)
EI change				0.034***		0.031***
				(0.003)		(0.003)
REShare change					$-$ 0.018***	$-$ 0.008***
					(0.003)	(0.002)
Constant	0.184***	0.316**	0.744***	0.360***	0.712***	0.385***
	(0.0683)	(0.125)	(0.151)	(0.111)	(0.132)	(0.107)
Country-fixed effects	No	Yes	Yes	Yes	Yes	Yes
Time-fixed effects	No	No	Yes	Yes	Yes	Yes
$R^2$	0.095	0.127	0.372	0.630	0.468	0.649
*N*	624	624	624	624	624	624

The dependent variable is the growth in per capita CO2 emissions for columns (1) to (6). The independent variables are the 1-year lagged level of per capita CO2 emissions (lag-CO2pc), the growth in per capita GDP (GDPpc growth), changes in energy intensity (EI ch.), and the change of the renewable share into the primary energy supply (RESharech). Standard errors are in parentheses. In column (1), we omit country- and year-fixed effects. (Constant can be dropped otherwise.) Country-fixed effects are introduced in column (2), and both of them are jointly considered in columns from (3) to (6). In column (4), we control for changes in EI; in column (5), for the renewable share change; and, finally, in column (6), we implement both kinds of controls. *$p < 0.10$, **$p < 0.05$, ***$p < 0.01$

With respect to our key parameter, $\beta _{2}$, it seems that the heterogeneity between countries, accounted for the corresponding fixed effects, drives only small differences in the estimates. This suggests that country unobservable heterogeneity is not key for the CO2–GDP elasticity. However, the magnitude of $\beta _{2}$ decreases with the inclusion of the time-fixed effect, which implies that part of the within-country correlation between CO2 and GDP growth is caused by factors that are common to all countries but time variant (eventually, the evolution of international oil prices, or changes in the European regulation), which affect both CO2 emissions and GDP growth along the business cycle. Precisely, this result is what we interpret in terms of the importance for CO2 emissions growth of the boom–bust cycle we observed in Western Europe associated with the 2000s expansion in emerging countries and the 2008 Great Recession afterward.^9^

This picture, however, is substantially modified once we incorporate the evolution of energy intensity in the regression: columns (4) and (6). In such a case, all estimates get back closer to the specification in (2), while the regression fit measured by the $R^2$ increases a lot: from 0.372 in column (3), to 0.630 in column (4). The idea is that the changes in energy intensity over the panel is the key variable to account for CO2 emissions growth, and at the same time, it controls for the business cycle and GDP growth. An additional interesting result is related to the potential bias reduction in the estimation of $\beta _{1}$ when energy intensity is incorporated to the model. As discussed above, pooled estimations of $\beta _{1}$ (column (1)) are upper biased (i.e., it get closer to zero), while estimations including fixed effects (column (3)) are downward biased. However, the inclusion of energy intensity changes in the model makes the estimated $\beta _{1}$ lie in between these previous estimates, which is an indicative of the bias reduction.

Changes in energy intensity have a positive and highly significant direct effect (short-run within-country correlation) on per capita CO2 emissions growth, but it also accounts (as a key driver) for the conditional convergence speed and, more importantly for our purposes, for the CO2–GDP elasticity. Its estimated coefficient is 0.0341 in column (4), while it suffers minor changes (its estimation is 0.0306) in column (6), when the changes of renewables are included. Recall that these coefficients are already adjusted by the standard deviation of the energy regressor. Hence, quantitatively, this result implies that an improvement (i.e., a reduction) in one standard deviation of $\Delta EI_{i,t}$ (equal to 16.7 kToe/BillionUSD, which represents about a 18% over the sample mean) is associated with a reduction between 3 and 3.4 p.p. in annual within-country per capita CO2 emissions in our sample. This quantity is meaningful as far as the standard deviation of per capita CO2 annual growth for the entire pool is 5.5 p.p.

Next, we evaluate the role of renewable energies with regressions (5) and (6). It is apparent, looking at column (5), that adding the changes in the share of renewables to the regression, without controlling for the changes in energy intensity, sends us back to the estimates in regression (3), possibly missing the economic boom and other common time-variant effects. More importantly, the estimated $\beta _{1}$ and $\beta _{2}$ are almost invariant with this inclusion as it is shown in column (5) and column (6). Thus, the role of changes in renewable use exhibits the right sign (its direct within-country effect on CO2 emissions is negative and highly significant) but has a moderate effect on the benchmark elasticities while abstracting from energy use. The estimated coefficient is $-0.018$ in column (5) and -0.0085 in column (6), when including also the changes in energy intensity. Quantitatively, the implication is that an increase in one standard deviation in the change of the renewable share (equal to 1.7 p.p. for the pool) is associated with a decrease in within-country CO2 emissions annual growth of about 1.8 p.p. or 0.85 p.p., depending on the estimated model. This quantity, although smaller than that found for EI, is of a relevant magnitude.

Overall, these results clearly illustrate on the evidence we want to stress in this paper. The fact that we can identify common slopes for Western European countries seems to help us to identify significant and negative climatic effects of expansionary economic behavior, which is still substantially driven by energy intensity. At the same time, improvements in carbon intensity have a limited role in counteracting such a negative transmission channel from economic activity to CO2 emissions growth. In the next section, we show how the CO2–GDP elasticity depends on the past position in energy intensity, while it does not on the lagged share of renewable energies.

### CO2–GDP elasticity and the energy technology

In this section, we focus on the interaction between the energy variables and the economic activity. This will allow us to capture the comovements between these groups of variables and analyze how the CO2–GDP within-country elasticity depends on the state of the energy technology, that is, on its energy intensity position and on its share of renewables energy. As discussed in Sect. 3, we consider two alternative crossed effects with per capita GDP growth: first, with respect to the lagged energy intensity position, $EI_{i,t-1} \cdot \Delta \ln Y_{i,t}$, and second, with the lagged share of renewables in the primary energy mix, $R_{i,t-1} \cdot \Delta \ln Y_{i,t}$.^10^ The question we want to address is whether the path of CO2 emissions for countries at different positions of their energy technologies is more or less responsive to business cycle fluctuations, measured here by their per capita GDP growth.

**Table 3:** Panel data estimates: CO2–GDP elasticity and energy issues

	CO2pc growth				
	(1)	(2)	(3)	(4)	(5)
Lag-CO2pc emissions	$-$ 0.0481***	$-$ 0.0447***	$-$ 0.0485***	$-$ 0.0476***	$-$ 0.0499***
	(0.0121)	(0.0121)	(0.0119)	(0.0119)	(0.0133)
GDPpc growth	0.100	0.744***	0.107	0.0591	$-$ 0.634
	(0.165)	(0.129)	(0.173)	(0.326)	(0.575)
EI change	0.031***	0.031***	0.031***	0.031***	0.031***
	(0.003)	(0.003)	(0.003)	(0.003)	(0.003)
REShare change	$-$ 0.008***	$-$ 0.008***	$-$ 0.008***	$-$ 0.008***	$-$ 0.007***
	(0.002)	(0.002)	(0.002)	(0.002)	(0.003)
GDPpc gr.$\times$ Lag$-$EI	0.137***		0.139***	0.146**	0.244**
	(0.0356)		(0.0353)	(0.0572)	(0.0949)
GDPpc gr.$\times$ Lag$-$RESh		0.0107	$-$ 0.0160	0.00811	0.137
		(0.0892)	(0.0893)	(0.0970)	(0.153)
Country-fixed effects	Yes	Yes	Yes	Yes	Yes
Time-fixed effects	Yes	Yes	Yes	Yes	Yes
$R^2$	0.654	0.649	0.654	0.653	0.631
*N*	624	624	624	610	560

The dependent variable is the growth in per capita CO2 emissions for columns (1) to (5). The independent variables are the 1-year lagged level of per capita CO2 emissions (lag-CO2pc), the growth in per capita GDP (GDPpc gr.), changes in energy intensity (EI ch.), and the change of the renewable share into the primary energy supply (RESh ch.). Two cross-effects are also considered: (i) the interaction between per capita GDP growth and the 1-year lagged level of EI (columns 1, 3–5), and (ii) between per capita GDP growth and the 1-year lagged level of the renewable share into the primary energy supply (columns 2, 3–5). All models control for country- and year-fixed effects. Column (4) excludes those countries accounting for the 1% highest or lowest GDPpc growth. We replicate this assessment in column (5), but considering the 5% of that measure. *$p < 0.10$, **$p < 0.05$, ***$p < 0.01$

Table 3 summarizes the estimation results when our baseline specification is augmented to incorporate these novel forms of interaction. All models in the table include country- and year-fixed effects, as well as the controls considered in (3.3). Column (1) includes the cross-effect between GDP growth and lagged EI, while column (2) includes the cross-effect with the lagged share of renewables. The rest of the columns include both terms, but in column (3), we use the entire sample, whereas, to avoid extreme volatile observations, in column (4) and (5) we exclude those country-year observations accounting for either the 1% or the 5% highest or lowest per capita GDP growth, respectively.

Compared to our reference estimates, the results now suggest that, on average, it is not at all GDP growth alone the key driver of CO2 emissions growth in Western Europe. Instead, it is GDP growth interacted with the lagged level of energy intensity. Actually, when including this interacted term, the variable GDP growth per se is no longer significant: its point estimate is just 0.10, and not-significant at usual levels.

Regression (2) in Table 3, in its turn, shows that when GDP growth is interacted with the lagged share of renewables energy nothing changes in the broad picture: The crossed term is non-significant, and the estimated CO2–GDP elasticity is almost the same than in the baseline specification in Table 2. Therefore, our data at this point are not supportive that any business cycle phenomena modify the role of renewables in mitigating CO2 emissions growth in Europe. This finding is possibly explained by the still low levels of renewables. Thus, even though we estimated that an increase in renewables’ share has a direct and beneficial effect on CO2 emissions, this is not enough, however, to modify the within-country CO2–GDP elasticity. Another explanation is moderate growth rates associated with the growth path in rich countries, where renewables are more present. For them, renewables shift downward the CO2–GDP schedule without modifying its slope.

We conclude that the different levels of energy intensity are the energy variable that may help to account for the observed heterogeneity in the CO2–GDP elasticity, whereas the potential role of renewable energies is not. According to our estimates, a country growing with low levels of energy intensity is able to almost perfectly decouple its economic growth from the generation of CO2 emissions. From there, differences of one standard deviation in energy intensity would be associated with nearly 0.14 points more in the CO2–GDP elasticity. The key policy implication of this finding is that a priority for the energy transition in Western Europe refers to reaching conditional convergence in energy intensity standards. This means each country has to balance its structural sectoral specialization with rationalizing the energy model and the allocation of energy-intensive industries toward given targets. In particular, conditional convergence in energy intensities may favor a bottom-up design of international climatic agreements according to which regional progresses in regulations expand and integrate to other countries as discussed in Battaglini and Harstad (2016).

To provide additional measures for the implied CO2–GDP elasticities, $\beta _{2}+\beta _{21}EI_{i,t-1}$, we evaluate this expression at different levels of EI. At the minimum level of EI in our sample, which is equal to 29.5 kToe/BillionUSD corresponding to Ireland in 2019, the implied CO2–GDP elasticity is 0.35. On the other extreme, evaluating this expression at the maximum level of EI in the pool (equal to 135.5 kToe/BillionUSD, associated with Denmark in 1980), we have that the resultant CO2–GDP elasticity is 1.23, which is more than triple the lower bound. Evaluating this statistic at the mean of EI in the pool (90.7 kToe/BillionUSD), the implied elasticity is 0.86, which is similar to the one we obtained from regression (6) in Table 2. Notice finally that this range of values, which goes from 0.35 to 1.23, is in line with the range of values provided in Table 1 when we showed the set of country-specific elasticities.

To further explore the energy intensity finding, we exclude from the sample some extreme value observations. First, in column (4), we drop out top and bottom 1% observations of per capita GDP growth. We find that it implies slightly less CO2 inertia, while the measurement of the role of the interaction between economic growth and CO2 levels goes up. No other estimate particularly changes. This specification involves only fourteen observations excluded that mostly belong to Ireland, a country with huge GDP growth and overly smooth emissions pattern over the sample. Finally, when we exclude extreme top and bottom 5% observations of GDP growth, the result reinforces the role of the interacted variable, while reducing the role of renewables at the same time that increases the explanatory power of the CO2 inertia.

### Energy and activity asymmetric effects

We have established that the observed heterogeneity in the response of CO2 emissions to economic activity in the short run is substantially driven by energy intensity. Moreover, the direct effect on carbon emissions of GDP growth seems actually captured by the interaction between GDP growth and the (lagged) level of energy intensity. In this section, we explore whether these findings respond to a potential asymmetric relationship between economic shocks, energy intensity, and CO2 emissions [e.g., Jaforullah and King (2017) and Wagner (2014), among others].

Our strategy here builds upon the application of the nonlinear autoregressive distributed lag model (ARDL) ideas [cf. Shin et al. (2014)]. Notice that the DPD specification in Eq. (3.3) is a particular panel ARDL model, including fixed and temporal effects. In particular, next we incorporate separately in the panel data two regimes: those where the explanatory variables exhibit either positive or negative changes. Thus, we explore the potential asymmetric effects by estimating two coefficients (increase and decrease) for each explanatory variable, that is, for per capita GDP growth, EI change, renewable share change, and the cross-effect.^11^

**Table 4:** Asymmetric effects of GDP growth and changes in energy intensity and renewable share on CO2 emissions growth

	CO2pc growth					
	(1)	(2)	(3)	(4)	(5)	(6)
Lag-CO2pc emissions	$-$ 0.0451***	$-$ 0.0450***	$-$ 0.0465***	$-$ 0.0471***	$-$ 0.0521***	$-$ 0.0543***
	(0.0123)	(0.0121)	(0.0125)	(0.0123)	(0.0121)	(0.0121)
GDPpc growth		0.753***	0.751***			
		(0.103)	(0.103)			
GDPpc growth$^{+}$	0.808***			0.808***	$-$ 0.117	$-$ 0.127
	(0.144)			(0.147)	(0.138)	(0.147)
GDPpc growth$^{-}$	0.608***			0.589***	1.446	1.377
	(0.193)			(0.203)	(0.957)	(0.963)
EI change	0.0306***		0.0301***		0.0316***	
	(0.00284)		(0.00280)		(0.00285)	
EI change$^{+}$		0.0308***		0.0310***		0.0325***
		(0.00653)		(0.00661)		(0.00670)
EI change$^{-}$		0.0304***		0.0294***		0.0301***
		(0.00437)		(0.00439)		(0.00437)
REShare change	$-$ 0.00846***	$-$ 0.00847***			$-$ 0.00788***	
	(0.00245)	(0.00245)			(0.00242)	
REShare change$^{+}$			$-$ 0.0144***	$-$ 0.0146***		$-$ 0.0141***
			(0.00403)	(0.00390)		(0.00387)
REShare change$^{-}$			$-$ 0.00182	$-$ 0.00159		$-$ 0.000953
			(0.00375)	(0.00380)		(0.00369)
GDPpc gr.$^{+}\times$ Lag$-$EI					0.206***	0.208***
					(0.0368)	(0.0369)
GDPpc gr.$^{-}\times$ Lag$-$EI					$-$ 0.179	$-$ 0.169
					(0.183)	(0.184)
Country-fixed effects	Yes	Yes	Yes	Yes	Yes	Yes
Time-fixed effects	Yes	Yes	Yes	Yes	Yes	Yes
$R^2$	0.649	0.649	0.653	0.654	0.658	0.663
*N*	624	624	624	624	624	624

The dependent variable is the growth in per capita CO2 emissions for columns (1) to (6). The independent variables are the 1-year lagged level of per capita CO2 emissions (lag-CO2pc), the growth in per capita GDP (GDPpc gr.), changes in energy intensity (EI ch.), and the change of the renewable share into the primary energy supply (RESh ch.). Asymmetric effects are built drawing on two dummies for each variable, one representing positive growths (i.e., GDPpc gr.$^{+}$, EI ch.$^{+}$ and RESh ch.$^{+}$) and the other one for negative growths (i.e., GDPpc gr.$^{-}$, EI ch.$^{-}$ and RESh ch.$^{-}$). Two cross-effects are also considered: the interaction between the 1-year lagged level of EI and asymmetric (positive and negative) per capita GDP growth (columns 5 and 6). All models control for country- and year-fixed effects. *$p < 0.10$, **$p < 0.05$, ***$p < 0.01$

Table 4 shows the estimated results. Columns (1) to (3) report the estimates case by case (i.e., considering the asymmetry for only one regressor at a time). Column (4) considers all asymmetries simultaneously, while columns (5) and (6) consider also asymmetries in the cross-term (the interaction between GDP growth and lagged EI). In general, we find that the estimated EI change coefficients are not different in their positive and negative regimes. In both regimes (column (2), (3), and (6)), the estimated coefficient is about 0.031 in most of the cases (the same coefficients than in Table 3). When the cross-effect is not included in the model, the CO2–GDP elasticity is higher for the positive GDP growing regime (about 0.81) than for the decreasing GDP regime (close to 0.60).

However, the most significant asymmetric effects are observed for the cross-effects and the share of renewables. When the cross-effect is included in the model, there is not statistically significance that GDP growth correlates with CO2 emissions growth in the regime where per capita GDP is falling (notice that this negative regime is associated with recessions).^12^ Rather, when GDP is increasing, which is the most common situation, the estimated results are similar than in the symmetric specification. With respect to the share of renewables, though, its coefficient turns almost zero (and non-significant) when the share is decreasing, but it almost duplicates its (negative) value when the share is increasing. In this latter regime, the estimated coefficient is about -0.014, which means that a one standard deviation increase in the share of renewables now is associated with a reduction in the annual within-country per capita CO2 emissions of about 1.4 p.p. (recall that reduction is about 0.85 p.p. in the symmetric case). We interpret these findings as an evidence that economic expansions are often amplified by energy-intensive sectors, and therefore, as an evidence that climate policy targets should be responsive to business cycle fluctuations.

Finally, we have also explored the possibility of common positive and negative regimes associated with each of the three regressors. We find evidence that periods when *EI* is shrinking (negative *EI* change) are associated with a stronger mitigating effect of the renewables share change. Likewise, periods when renewables’ share is shrinking are associated with a stronger polluting effect of GDP growth. This finding has the clear policy implication of a macroeconomic and energy policy coordination. We will get back to this issue in Sect. 5.2.

In this section, the focus has been mostly on the time dimension of the panel. Next, we perform a series of robustness checks that exploit relatively more the country-specific dimension of the panel.

## Robustness checks

Next, we present four alternative specifications for the within-country short-run estimates, here aiming to address the issues of (i) the particular role of different countries, (ii) the precise role of boom–bust dynamics, (iii) the precise role of different renewable energies, and (iv) a preliminary account for the role of foreign trade. We examine these four alternative specifications in turn, and we show that in all of the cases our benchmark regression coefficients remain plausible.

### The role of particular countries.

In Sect. 2 we explored the general evidence which is the focus in this paper, both in the data pool and country by country. There we established the key sources of heterogeneity, and the way in which the energy intensity variable was able to account for a lot of that heterogeneity. With the panel estimates we think we have constructed the adequate metric. One may think, however, that the average results in the panel for the income elasticity of CO2 emissions depend on the influence of particular countries. Actually, it is for this reason that in Table 3, we discussed the results when excluding the top and bottom 1% or 5% country-years observations.

Rather, the results in Table 5 go back to our benchmark panel regression in Sect. 4.2, but now excluding one by one the effect of a particular country in the sample. Each particular case for the estimates can be traced to the comparable result obtained in the pool for the 16 Western European countries (WE-16). The benchmark regression, as in Sect. 4, includes the growth in per capita GDP (GDPpc growth), the 1-year lagged effect of per capita CO2 emissions (L1.CO2pc), and the crossed effect between growth in per capita GDP and the 1-year lagged level of energy intensity (EI). The model also controls for changes in EI and the renewable share into the primary energy supply, and both for time- and year-fixed effects. The indicated country in the table is excluded at every alternative specification.

**Table 5:** Robustness analysis: country impact on estimates

	GDPpc Growth		Lag-CO2pc		Cross-effect	
	Coefficient	Std. Error	Coefficient	Std. Error	Coefficient	Std. Error
WE16	0.1004	0.1654	$-$ 0.0481	0.0121	0.1374	.0356
Austria	0.0902	0.169	$-$ 0.0491	0.0124	0.1398	.0362
Belgium	0.1283	0.1656	$-$ 0.0467	0.0123	0.1332	.0358
Denmark	0.1282	0.17	$-$ 0.0578	0.0122	0.1124	0.0366
Finland	0.0459	0.1644	$-$ 0.0438	0.0123	0.1491	0.0365
France	0.1381	0.1692	$-$ 0.0419	0.0127	0.129	0.0361
Germany	0.0985	0.1701	$-$ 0.0516	0.0129	0.134	0.0367
Greece	0.0742	0.171	$-$ 0.0453	0.0131	0.1485	0.0381
Ireland	$-$0.0247	0.5746	$-$ 0.0481	0.0130	0.1618	0.0996
Italy	0.1081	0.1723	$-$ 0.0477	0.0122	0.1359	0.0369
Netherlands	0.0647	0.1639	$-$ 0.0479	0.0122	0.1508	0.0353
Norway	0.156	0.1604	$-$ 0.0436	0.0117	0.1324	0.035
Portugal	0.0905	0.1589	$-$ 0.0578	0.0138	0.1359	0.0353
Spain	0.1585	0.1725	$-$ 0.0518	0.0127	0.1237	0.037
Sweden	0.1119	0.1651	$-$ 0.042	0.0119	0.1419	0.0354
Switzerland	0.102	0.1721	$-$ 0.0463	0.0123	0.1392	0.0367
UK	0.0797	0.1722	$-$ 0.0514	0.0126	0.1394	0.0375

The dependent variable is the growth in per capita CO2 emissions. The independent variables are the growth in per capita GDP (GDPpc growth), the 1-year lagged effect of per capita CO2 emissions (Lag CO2pc), and the cross-effect between growth in per capita GDP and the 1-year lagged effect of energy intensity (EI). The model also controls for changes in EI, the growth of the renewable share into the primary energy supply, and both time- and year-fixed effects. One country is excluded at every time. Coefficients for GDPpc growth are non-significant (due to the inclusion of the cross-effect), while both those of Lag-CO2pc and the cross-effect are significant at 1% level

The first result is that the coefficients for per capita GDP growth are non-significant, as they were in our benchmark regression in Table 3. On the other hand, with respect to the inertia term of the regression (Lag CO2pc, significant at 1% level) we observe that the estimate in the pool ($-0.048$) is not significantly different of that obtained in all the other particular cases, although excluding either Denmark or Portugal produces the faster convergence result ($-0.058$), with an intermediate result while excluding Germany, Spain, or the UK ($-0.052$). That is, excluding those particular cases, one estimates less CO2 emissions growth the higher the starting levels. Finally, the key driver is in the crossed effect between growth in per capita GDP and the 1-year lagged effect of energy intensity (Lag-EI). The average estimate is 0.137, and again, we do not estimate significant differences by excluding particular countries. However, the stronger effect on CO2 emissions of the interaction between growth and energy intensity is more important when we exclude Ireland indeed, a country growing a lot in the sample with a relatively low energy intensity. That is, including Ireland in the pool contributes to a more favorable to growth overall picture: less importance of growing with energy intensity. This circumstance occurs to a lesser extent when excluding the Netherlands, Finland, or Sweden (relatively low energy intensity, also with growth), but also with Greece (the other way around). On the contrary, excluding Denmark or Spain reverts the picture, that is, having them increased the importance of the crossed effect. We think this as an important evidence to keep in mind. Something that we can call “polluting when growing like Spain (or Denmark).”

### The boom and the bust

Of particular incidence for the analysis might be the boom we observed in Europe at the peak of the global industrial cycle in emerging countries along the 2000s, and the bust that arrived with the Great Recession afterwards. We discussed above a strong evidence of business cycle patterns associated with CO2 emissions growth. It can be argued that the within-country pattern we found in the panel is clearly more present in some countries than others, as far as the boom–bust cycle is not the same everywhere.

**Table 6:** CO2–GDP elasticity under alternative growth rates regimes

	CO2pc growth	
	(1)-low	(2)-high
Lag-CO2pc emissions	$-$ 0.0648***	$-$ 0.0496***
	(0.0172)	(0.0134)
GDPpc growth	0.570	$-$ 0.376*
	(0.784)	(0.216)
EI change	0.125***	0.037***
	(0.0225)	(0.003)
REShare change	$-$ 0.0423**	$-$ 0.012***
	(0.0214)	(0.053)
GDPpc gr.$\times$ Lag$-$EI	$-$ 0.0254	0.276***
	(0.149)	(0.0530)
Country-fixed effects	Yes	Yes
Time-fixed effects	Yes	Yes
$R^2$	0.616	0.770
*N*	312	312

The dependent variable is the growth in per capita CO2 emissions for both columns. The independent variables are the 1-year lagged level of per capita CO2 emissions (lag-CO2pc), the growth in per capita GDP (GDPpc gr.), changes in energy intensity (EI ch.), and the change of the renewable share into the primary energy supply (RESh ch.). A cross-effect between per capita GDP growth and the 1-year lagged level of EI is also considered. All models control for country- and year-fixed effects. In column (1), only country-years observations that account for a per capita GDP growth lower than the median of the sample are considered. The results corresponding to the opposite case are reported in column (2). *$p < 0.10$, **$p < 0.05$, ***$p < 0.01$

Therefore, an alternative strategy we follow next is to split the sample between country-year observations under a low GDP growth versus a high GDP growth regime. We test this new hypothesis on our benchmark regression specification which includes the energy intensity interaction. We assign a country-year observation to the low-growth regime if it corresponds to a per capita GDP growth over the period lower than the median GDP growth in the sample, and the opposite for the high-growth regime. This will illustrate on the relative importance for each of the two groups of the highlighted business cycle aspect of the within-country estimates.

Table 6 reports these particular results. Column (1) shows that GDP growth, neither per se nor interacted with the 1-year lagged level of energy intensity (Lag-EI), is a significant variable to account for CO2 emissions growth among the low-growth regime observations. Rather, it is CO2 emissions inertia (L1.CO2pc), and overall, the changes in energy intensity ($\hbox {EI}_{\text{ ch }}$) the variables that clearly determine the pattern of CO2 emissions growth, precisely: ${\widetilde{\beta }}_1^{(1)} = -0.0648$ vs. ${\widetilde{\beta }}_1^{(2)} = -0.0496,$ and notably, ${\widetilde{\beta }}_3^{(1)} = 0.125$ vs. ${\widetilde{\beta }}_3^{(2)} = 0.037$. At the same time, column (2) in Table 6 for the high growth observations highlights the importance of the transmission of GDP growth to CO2 emissions through the interacted term with the 1-year lagged level of energy intensity (Lag-EI), ${\widetilde{\beta }}_5^{(2)} = 0.276$. This interacted effect takes over the direct effect, which changes sign, and it is only significant at a 10% level. The implication is, again, that countries with high levels of energy intensity in the past that experience a boom (bust) will be expected to give rise to sizeable increases (decreases) in carbon emissions. This confirms this particular feature of the data as something to take into account when designing the EU stimulus package for the recovery from the Covid-19 crisis.

Finally, the beneficial effect on CO2 emissions of the changes in the share of renewable energies in the primary mix is more important in the low-growth country-years observations, although it is also subject to a higher variability: The estimate here is at a 5% significance level. Again, the level of the renewable share variable might be playing for the variability in the size of the estimated effect. This leads us to further explore the role of renewables.

**Fig. 4:**
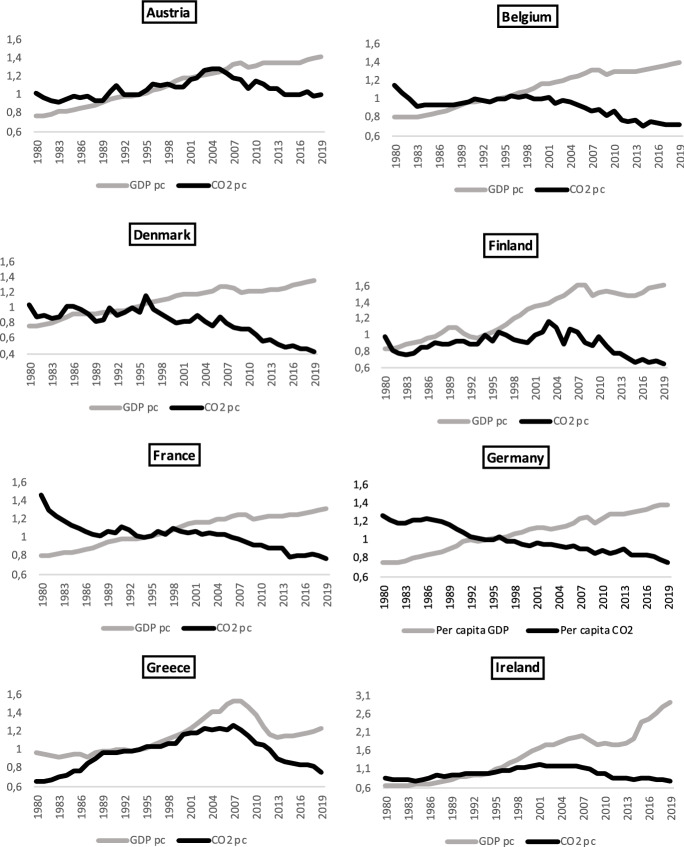

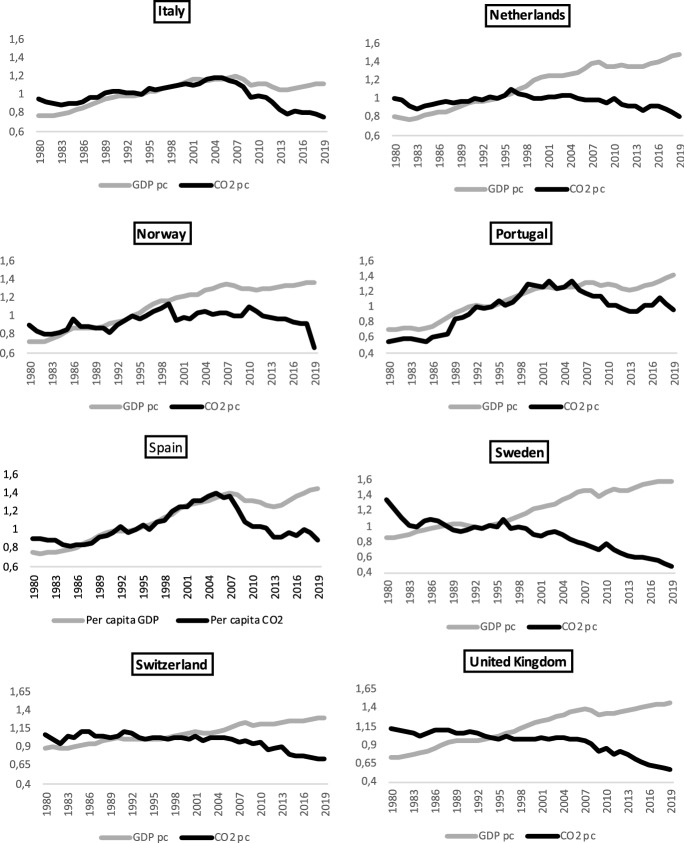
Per capita GDP and per capita CO2 indexes between 1980 and 2019 (index $1994=1$)

**Fig. 5:**
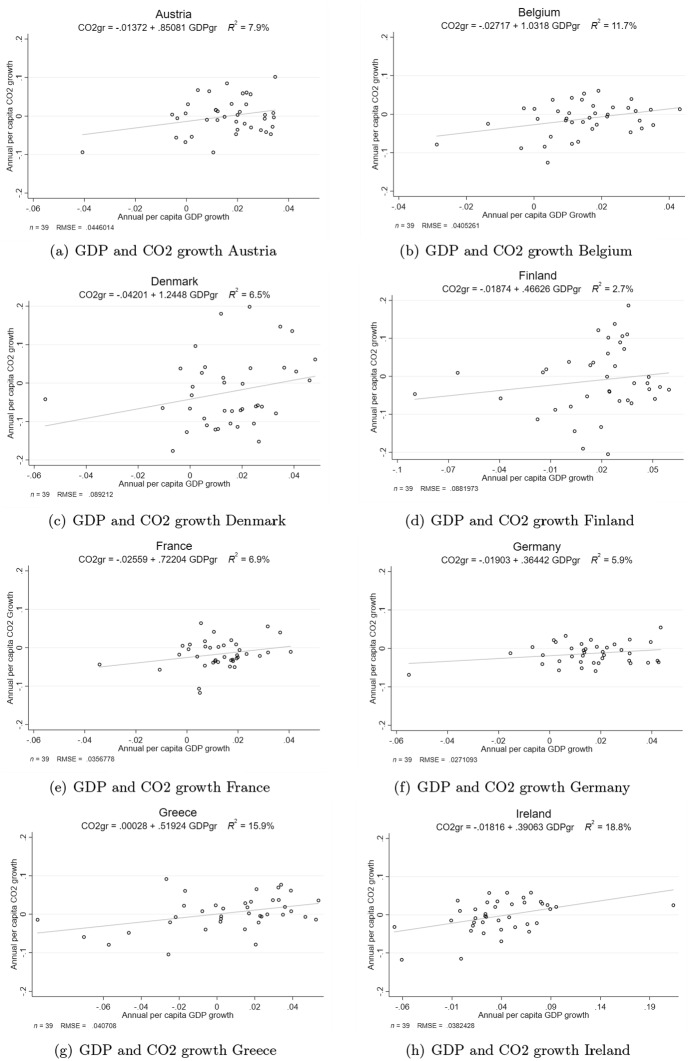

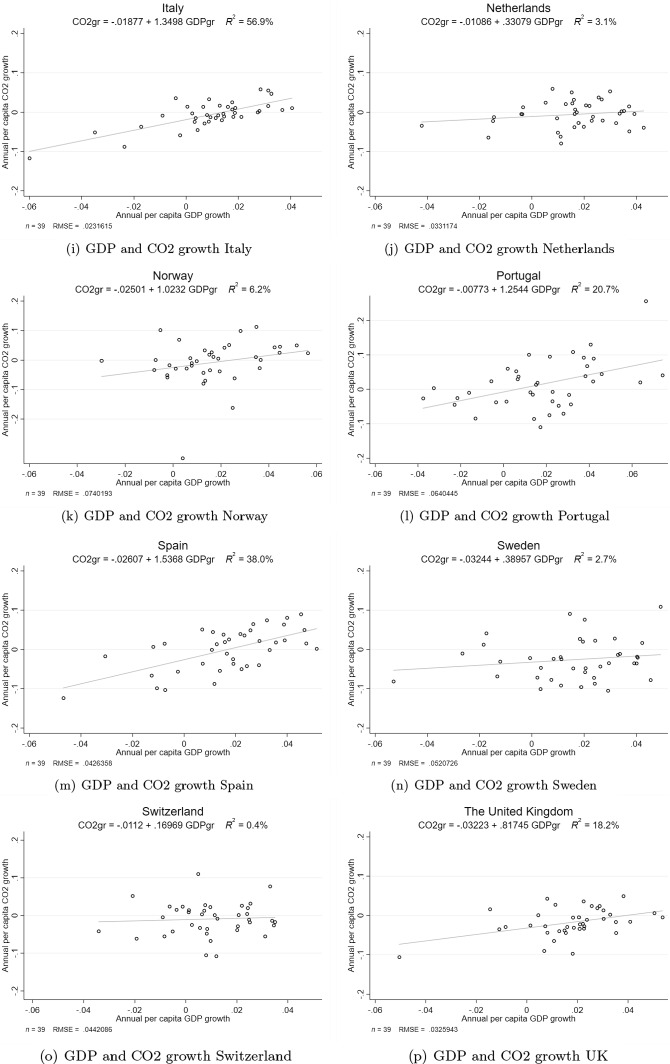
Per capita CO2 growth and per capita GDP growth between 1980 and 2019

**Fig. 6:**
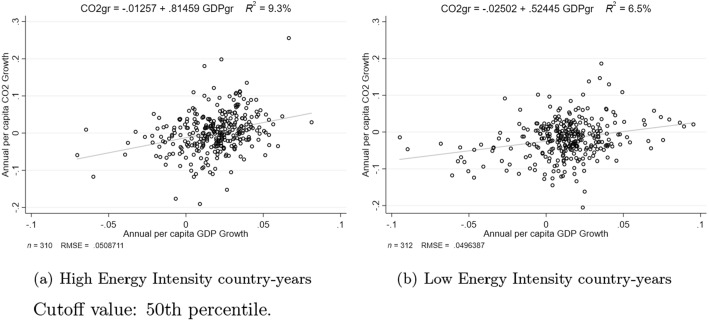
CO2–GDP elasticity: **a** High EI country years. **b** Low EI country years. Cutoff value: 50th percentile

**Fig. 7:**
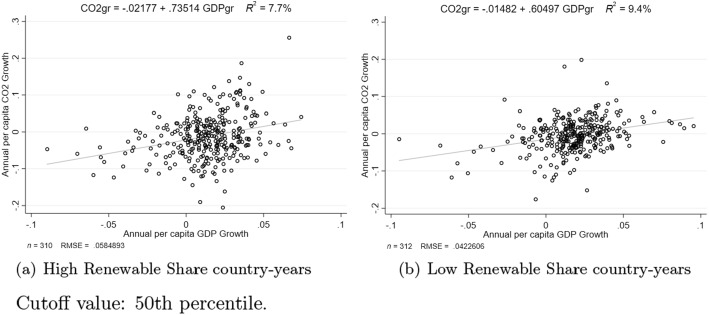
CO2–GDP elasticity: **a** High RS country years. **b** Low RS country years. Cutoff value: 50th percentile

We further explore the boom–bust result while in addition considering the asymmetric effects discussed above. Rather than splitting the sample between low and high country-year observations, we now distinguish directly between countries. Precisely, we separate those countries that seem to have been decoupling carbon emissions from economic growth in our sample period. When emissions decrease relative to economic growth, we speak of (absolute) decoupling. This situation is illustrated in Fig. 4 in “Appendix B,” from which we identify those countries with a negative comovement between per capita CO2 and GDP levels for most of the last forty years. Visual inspection of the data identifies Belgium, France, Germany, the Netherlands, Sweden, Switzerland, and the UK as countries in the decoupling set.^13^

**Table 7:** Panel data estimates: Decoupling countries versus non-decoupling countries

	CO2pc growth					
	(1)	(2)	(3)	(4)	(5)	(6)
Lag-CO2pc emissions	$-$ 0.0443***	$-$ 0.0422***	$-$ 0.0407***	$-$ 0.0385***	$-$ 0.0467***	$-$ 0.0410***
	(0.0124)	(0.0119)	(0.0119)	(0.0119)	(0.0130)	(0.0125)
GDPpc growth		0.765***	0.749***			
		(0.101)	(0.0984)			
GDPpc growth$^{dec.}$	0.674***			0.628***	0.0981	0.0717
	(0.159)			(0.152)	(0.624)	(0.600)
GDPpc growth$^{non-dec.}$	0.764***			0.776***	0.0924	0.153
	(0.106)			(0.103)	(0.168)	(0.169)
EI change	0.0305***		0.0307***		0.0310***	
	(0.00285)		(0.00284)		(0.00285)	
EI change$^{dec.}$		0.0242***		0.0257***		0.0260***
		(0.00296)		(0.00303)		(0.00310)
EI change$^{non-dec.}$		0.0334***		0.0328***		0.0333***
		(0.00355)		(0.00354)		(0.00352)
REShare change	$-$ 0.00850***	$-$ 0.00800***			$-$ 0.00814***	
	(0.00245)	(0.00244)			(0.00244)	
REShare change$^{dec.}$			0.00133	$-$ 0.000533		$-$ 0.000795
			(0.00341)	(0.00336)		(0.00341)
REShare change $^{non-dec.}$			$-$ 0.0110***	$-$0.0101***		$-$ 0.00963***
			(0.00302)	(0.00299)		(0.00297)
GDPpc gr.$^{dec.}\times$ Lag-EI					0.119	0.114
					(0.113)	(0.108)
GDPpc gr.$^{n-d.}\times$ Lag-EI					0.142***	0.132***
					(0.0355)	(0.0354)
Country-fixed effects	Yes	Yes	Yes	Yes	Yes	Yes
Time-fixed effects	Yes	Yes	Yes	Yes	Yes	Yes
$R^2$	0.649	0.654	0.657	0.660	0.654	0.664
*N*	624	624	624	624	624	624

The dependent variable is the growth in per capita CO2 emissions for columns (1) to (6). The independent variables are the 1-year lagged level of per capita CO2 emissions (Lag-CO2pc), the growth in per capita GDP (GDPpc gr.), changes in energy intensity (EI ch.), and the change of the renewable share into the primary energy supply (RESh ch.). Additionally, variables for decoupling (dec.) and non-decoupling (non-dec./n-d.) countries are built from one dummy each. Two crossed effects are also considered: the interaction between the 1-year lagged level of EI and per capita GDP growth for decoupling and non-decoupling countries (columns 5 and 6). All models control for country- and year-fixed effects. *$p < 0.10$, **$p < 0.05$, ***$p < 0.01$

Table 7 reports these complementary results. First, inertia under this specification is slightly lower than in the benchmark regression with asymmetries in Table 4. Second, GDP growth makes little difference but clearly, energy intensity (*EI*) and renewable share changes weigh more for non-decouplers (columns (1), (2) and (3)). All decouplers/non-decouplers asymmetries considered (column (4)) produce the same result. Likewise, incorporating the cross-effect displays the same pattern than in Table 3. Finally, the cross-effect jointly with all the decoupling asymmetry retains both the importance of *i)* the interaction: the effect of GDP whenever *EI* is high, and *ii)* the mitigating role of increases in renewable share, for non-decouplers

### Anatomy of renewables

At this point, the carbon intensity component of the CO2 emissions conundrum, the one that should be related to the use of clean energy, and thus, with an increased share of renewable energies, is not showing a vigorous stance. It can be argued that not all changes in the different renewable energies are alike in the pool. Next, we consider not only the change of the renewable share into the primary energy supply, but also the change in the shares corresponding to solar photovoltaics (PVShare change), solar thermal (THShare change) and wind (WindShare change) energies.

Table 8 reports the results with and without interacted terms. The key observation is the stability of the renewable share change estimate ($\hbox {RES}_{\text{ share }}$ change) with ${\widetilde{\beta }}_{4}$ at a value of -0.012 in all of the cases, as it was, for instance, the value in the case of the high-growth regime above. However, the variable that is taking the lead in terms of the beneficial effect of renewables is the change in the share of wind energy, a coefficient in line with the low-growth countries estimate above. This is an expected result, but the evidence we bring about in this paper gives a measure of the quantitative importance of this instrument of the green transition in Europe.

Otherwise, column (4) in Table 8 preserves our benchmark estimates with a direct effect of EI changes at about 0.03 and an interacted effect of 0.1 (remember, jointly, an improvement in one standard deviation of $\Delta EI_{i,t}$ is associated with a reduction of two-thirds of a standard deviation of CO2 emissions growth), and also, the inertia component of per capita CO2 emissions (L1.CO2pc) and the limited role of GDP growth per se ($\hbox {GDP}_{\text{ pc }}$ growth).

### Outsourcing emissions (or not)

Openness to trade among these countries is an important issue, as it is a well-recognized feature in a globalized world there is always the option to outsource CO2 emissions. This is a complicated matter which goes beyond carbon leakage and that may involve producing abroad in energy-intensive sectors, but also various forms of sectoral reallocation or even taking the lead in exporting fossil fuels. We leave these issues for further research.

However, there is a simple exploration related to the potential role of oil imports. We consider this new variable also taken from the IEA (2020). We find that the correlation between the changes in energy intensity and the changes in oil imports is positive and very high, even when we control for fixed effects. This means that energy intensity seems associated with energy dependence, another important issue for policy consideration. However, when we control for the changes in energy intensity in the panel regression, the fact that some countries import oil in a bigger share over their GDP does not change the broad picture of our within-country estimates. That is, most of the action is still in the dynamics of energy intensity and with its interaction along a booming economy.

**Table 8:** Panel data estimates: CO2–GDP elasticity and energy issues: detail of renewables

	CO2pc growth			
	(1)	(2)	(3)	(4)
Lag-CO2pc emissions	$-$ 0.0429***	$-$ 0.0454***	$-$ 0.0426***	$-$ 0.0454***
	(0.0122)	(0.0121)	(0.1218)	(0.0120)
GDPpc growth	0.7876***	0.2896*	0.7599***	0.2881*
	(0.0975)	(0.1725)	(0.1174)	(0.1814)
EI change	0.0331***	0.034***	0.033***	0.034***
	(0.003)	(0.003)	(0.003)	(0.003)
REShare change	$-$ 0.012***	$-$ 0.012***	$-$ 0.012***	$-$ 0.012***
	(0.002)	(0.002)	(0.002)	(0.002)
GDPpc gr.$\times$ Lag$-$EI		0.1062***		0.1058***
		(0.0392)		(0.0381)
GDPpc gr.$\times$ Lag$-$RESh			0.0314	0.0036
			(0.0859)	(0.0842)
PVShare ch.	0.050	0.056	0.049	0.056
	(0.038)	(0.037)	(0.038)	(0.037)
THShare ch.	$-$ 0.002	$-$ 0.002	$-$ 0.002	$-$ 0.002
	(0.033)	(0.033)	(0.033)	(0.033)
WindShare ch.	$-$ 0.046***	$-$ 0.040**	$-$ 0.047***	$-$ 0.040***
	(0.015)	(0.016)	(0.015)	(0.016)
Country-fixed effects	Yes	Yes	Yes	Yes
Time-fixed effects	Yes	Yes	Yes	Yes
$R^2$	0.727	0.729	0.727	0.729
*N*	591	591	591	591

The dependent variable is the growth in per capita CO2 emissions for columns (1) to (4). The independent variables are the 1-year lagged level of per capita CO2 emissions (lag-CO2pc), the growth in per capita GDP (GDPpc gr.), changes in energy intensity (EI ch.), and the change of the renewable share into the primary energy supply (RESh ch.), but also the change in the shares corresponding to solar photovoltaics (PVShare ch.), solar thermal (THShare ch.) and wind (WindShare ch.) energies. Two cross-effects are also considered: (i) the interaction between per capita GDP growth and the 1-year lagged level of EI (columns 2 and 4) and (ii) between per capita GDP growth and the 1-year lagged level of the renewable share into the primary energy supply (columns 3 and 4). All models control for country- and year-fixed effects. *$p < 0.10$, **$p < 0.05$, ***$p < 0.01$

## Conclusion

In this paper, we explore the transmission channels from economic activity toward CO2 emissions. We do so for a set of Western European countries that share in common important aspects of their production and energy technologies. For these countries, we estimate the short-run within-country CO2–GDP elasticity by using dynamic panel data methods. Our empirical implementation builds upon a neoclassical theoretical framework with energy use and emissions.

We show that a key channel in the aforementioned transmission is through the dynamics of energy intensity. We find that reductions in energy intensity are the action with the more important beneficial effects for the positive evolution of CO2 emissions growth in the group of countries we consider. Moreover, economies that have exhibited high energy intensity levels in the recent past produce much increased CO2 emissions during an economic boom. This feature of the data should be taken into account when designing the climate transition goals targeted in the EU’s Recovery Plan, whose main objective at this very moment is to mitigate the economic and social impact of the coronavirus pandemic. Also, this policy design issue is particularly relevant as far as we find in this paper that increases in the share of renewable energies in the primary energy supply seem to have a very moderate effect on the within-country, short-run CO2–GDP elasticities. Notwithstanding, the mitigating direct effect of a gain in the renewable share of the primary mix is particularly important whenever the energy intensity level is high and the economy is experiencing a boom.

The main implication of all these findings is the absolute priority for policies that contribute to reaching conditional convergence in energy intensity standards across Western European countries. Structural sectoral specialization across countries has to be balanced with rationalizing the energy model and the allocation of energy-intensive sectors across the European Union. Clearly though, structural change is not a policy instrument, so global convergence is not realistic. It is for this reason that we elaborate on incorporating short-run cyclical concerns into climate policy. These may come in the form of procyclical fuel taxes and fuel economy standards in the transport sector, as well as procyclical regulations toward energy efficiency and inducement for renewable energies in the power sector. It has been proposed the use of a policy thermostat to smooth the costs of climate policy to given targets. Our findings suggest that policy instruments might incorporate cyclical adjustment mechanisms while providing carbon price predictability.

Finally, gradualism in the climate transition goals targeted in the EU’s Recovery Plan and a focus on energy efficiency within each sector should be expected to offer a better return in favor of absolute decoupling in the short-to-medium run. The fact is that decoupling economic growth from CO2 emissions in Western Europe requires primarily the reduction in the levels of energy intensity within each sector in every country.

## The data

In this Appendix, we document the construction of the data series we use and some basic evidence we use to develop the argument in the main text. First, we provide a description of the data sources and the definition of all variables (see Table 9). Second, in Table 10 we report the main descriptive statistics, which are briefly discussed along Section 2.1. Finally, we report a basic evidence on the correlation of annual per capita CO2 growth with, respectively, CO2 levels in 1980 (see Fig. 3a) and annual per capita GDP growth (see Fig. 3b), both discussed in Sect. 2.1 as well.

**Table 9:** Data sources and description

Variable	Description	Source
GDP	Gross Domestic Product measured as Power Purchasing Parities (PPPs) at 2015 in billion USD	
Population	Number of inhabitants in millions	
GDPpc	Per capita GDP. GDP to population ratio measured in 2015 USD per capita	
TES	Total (primary) Energy Supply measured in million tonnes of oil equivalent	
EI	Energy Intensity. TES to GDP ratio measured in toe per thousands of USD	IEA’s World Energy Balances
RES share	Share of renewable energies into the primary energy supply measured as percentage	2020 Database (i.e., in TES)
PV share	Share of photovoltaics into the primary energy supply (i.e., in TES) measured as percentage	
TH share	Share of solar thermal energy into the primary energy supply (i.e., in TES) measured as percentage	
Wind share	Share of wind energy into the primary energy supply (i.e., in TES) measured as percentage	
CO2	Total CO2 emissions from fuel combustion measured in thousands of tonnes of CO2. Among others, it includes emissions related to the following sectors: industrial, residential, agriculture, fishing, heat and electricity production	
CO2pc	Per capita CO2 emissions. CO2 to population ratio measured in tonnes of CO2 per one thousand of inhabitants	IEA’s CO2 Emissions
CI	Carbon Intensity. CO2 to TES ratio measured in tonnes of CO2 per thousands of tonnes of oil equivalent	2020 Database

**Table 10:** Per capita CO2 emissions and per capita GDP sample statistics in WE16 countries

	CO2pc 1980	CO2pc 2019	CO2pc growth	GDPpc 1980	GDPpc 2019	GDPpc growth
WE16 Pool	8.330	5.719	$-$ 0.96	26.502	49.567	1.61
Austria	7.199	7.067	$-$ 0.05	28.800	52.853	1.56
Belgium	12.772	7.892	$-$ 1.23	27.574	48.300	1.44
Denmark	12.317	4.937	$-$ 2.34	29.565	52.900	1.49
Finland	11.478	7.520	$-$ 1.08	23.755	45.980	1.69
France	8.253	4.347	$-$ 1.64	26.279	43.072	1.27
Germany	13.386	7.933	$-$ 1.34	27.496	50.143	1.54
Greece	4.638	5.306	0.34	22.544	28.596	0.61
Ireland	7.616	6.773	$-$ 0.30	18.582	84.312	3.88
Italy	6.297	5.015	$-$ 0.58	26.602	38.652	0.96
Netherlands	10.277	8.231	$-$ 0.57	29.346	53.952	1.56
Norway	6.659	4.868	$-$ 0.80	32.499	62.056	1.66
Portugal	2.409	4.264	1.46	16.513	33.101	1.78
Spain	4.904	4.880	$-$ 0.01	19.717	38.093	1.69
Sweden	8.799	3.157	$-$ 2.63	27.307	50.569	1.58
Switzerland	6.144	4.234	$-$ 0.96	45.340	66.282	0.97
UK	10.129	5.076	$-$ 1.77	22.119	44.212	1.78

Per capita GDP is measured in thousands of USD, using 2015 PPPs. Per capita CO2 is measured in tons per capita. Growth is referred to the average yearly growth

## Basic CO2–GDP relationship, all countries

In this Appendix, we report for the 16 Western European (WE16) countries the time series behavior of per capita CO2 emissions and per capita GDP. The corresponding discussion is in the main text (Fig. 4).

We also report the relationship between annual per capita CO2 growth and per capita GDP growth for all the 16 Western European Countries. The corresponding discussion is in the main text (Fig. 5).

## Basic CO2-Energy relationship, all countries

In this Appendix, we report some additional facts on the CO2 emissions–energy relationship. Figure 6 reports the CO2–GDP elasticity for high energy intensity country years, and for low energy intensity country years, where the cutoff value is the 50th percentile. Correspondingly, Fig. 7 reports the CO2 emissions–GDP elasticity for high renewable share country years, and for low renewable share country years. Again, the cutoff value is the 50th percentile. The corresponding discussion is in the main text.

## Footnotes

1 This might be the case in Spain or the UK more than in Germany, for instance. The hypothesis has been explored for Spain vs Germany in Guinea et al. (2019), following Beaudry and Portier (2006) or Jaimovich and Rebelo (2009), among others. For a detailed description of the patterns observed for energy-intensive sectors during the Spanish housing boom of the 2000s, see Gutiérrez et al. (2011).

2 In these scatter charts, we see a large outlier corresponding to the growth rate in 2009 (the beginning of the Great Recession). Controlling for this anomaly almost does not affect the CO2–GDP elasticity for Spain and significantly reduces that of Germany (down to 0.08).

3 In fact, year 2009 is an anomaly in almost all countries. However, only in some cases such an outlier has a significant effect. This occurs for Austria, Belgium, and the UK, whose elasticities are lower, and Denmark, for which it increases significantly. Of all these, the case of Belgium should be highlighted, since it is the presence of this anomaly that makes its elasticity greater than one.

4 As in Stokey’s model, we deal with environmental pollution as proportional to production, where the use of increasingly clean techniques reduces the pollution/output ratio. This is related to Hassler et al. (2020).

5 An additional concern might be associated with potential uncertainty in variable selection. Again, the various techniques to address this issue are designed either for large cross sections or when it is necessary to retain only a subset of the regressors. We believe that our theoretical model supports the parsimonious specification of the empirical exercise we propose for our data.

6 In this specification, as discussed above, we are implicitly assuming that $P_{i,t}=(1+\beta _{1})\,P_{i,t-1}$, and this is the reason we associate a negative elasticity $\beta _{1}$ to reflect convergence in CO2 emissions across countries. Notice that the parameter $\beta _{1}$ is not directly the speed of convergence. In this setting, the speed of convergence is defined as ${\widetilde{\beta }} =-\ln (1+\beta _{1})$. An alternative way to measure the speed of convergence is using the half-life, i.e., the time required by a country to cover a half of the distance to their own steady state, which is given by $-\ln (2)/\ln (1+\beta )$.

7 We have considered the lagged of the saving rate and the lagged trade-weighted world income, as proposed by Acemoglu et al. (2008) as instruments for real per capita GDP to characterize its relationship with democracy, but we obtain unsatisfactory results in our case, as expected.

8 Related results for a much bigger cross section (actually, worldwide) by using system-GMM are discussed in Díaz et al. (2019).

9 Notice the short-term approach corresponding to our empirical model specification in the growth rates of variables renders GDP square non-significant. The Environmental Kuznets Curve (EKC) hypothesis is not key in our sample.

10 We also analyze the interaction between GDP growth and past emissions levels, that is $\ln P_{i,t-1}\Delta \ln Y_{i,t}$, but the interaction term is non-significant, and all other estimation results do not change. These particular results are available upon request.

11 An alternative strategy to analyze these asymmetries would be to specify and estimate an asymmetric panel ARDL, which, indeed, is not a trivial extension of this approach. However, this alternative prevents the results be compared with our previous (and posterior) dynamic panel data estimates.

12 The estimated coefficient for GDP growth when GDP is falling is well above one, but not significant. This implies that a reduction in GDP is partially correlated with reductions in carbon emissions, but the evidence suggests a high dispersion in that relationship.

13 Denmark and Finland may exhibit this pattern after 1995, and Ireland after 2000.
